# Actors influencing cancer-related fatigue and the construction of a risk prediction model in lung cancer patients

**DOI:** 10.3389/fonc.2024.1485317

**Published:** 2025-01-24

**Authors:** Mei-Ning Zhang, Yi-Chen Zhou, Zhu Zeng, Cun-Liang Zeng, Bo-Tao Hou, Gui-Rong Wu, Qiao Jiao, Dai-Yuan Ma

**Affiliations:** ^1^ Nursing Department, Dazhou Central Hospital, Dazhou, Sichuan, China; ^2^ Department of Oncology, Dazhou Central Hospital, Dazhou, Sichuan, China; ^3^ Cardiac Vascular Surgery, Dazhou Central Hospital, Dazhou, Sichuan, China; ^4^ Department of Oncology, Dazhou Integrated Traditional Chinese Medicine (TCM) and Western Medicine Hospital, Dazhou, Sichuan, China; ^5^ Department of Oncology, Affiliated Hospital of North Sichuan Medical College, Nanchong, Sichuan, China

**Keywords:** lung cancer, cancer-related fatigue, sleep quality, anxiety, depression, risk prediction model, logistic regression analysis

## Abstract

**Purpose:**

The paper aims to investigate the factors influencing cancer-related fatigue (CRF) in lung cancer patients and construct a CRF risk prediction model, providing effective intervention strategies for clinical medical staff.

**Methods:**

This paper employs convenience sampling to select 400 lung cancer patients who visited a tertiary hospital in Dazhou, Sichuan Province, from January 2021 to January 2022. A questionnaire survey was conducted using the Revised Piper Fatigue Scale (PFS-R), Pittsburgh Sleep Quality Index (PSQI), and Hospital Anxiety and Depression Scale (HADS) to collect data on patient demographics and sociological characteristics, disease-related information, physiological indicators, sleep quality, mental health, and other relevant factors. To explore the factors influencing CRF in lung cancer patients, single-factor analysis and multiple logistic regression analysis were performed. A CRF risk prediction model was then established, with its predictive performance and calibration evaluated using ROC curves.

**Findings:**

The results of multivariate logistic regression analysis showed that gender, age, education level, living status, daily exercise, clinical stage, course of disease, treatment mode, chronic disease, BMI, hemoglobin, serum albumin, blood glucose, potassium concentration, magnesium concentration, PSQI score and HAD score were the influencing factors of CRF in lung cancer patients (P<0.05). The AUC of the model construction group and the model validation group were 0.863 and 0.838, respectively, and the results of Hosmer-Lemeshow fit test showed that χ^2^ = 7.540, P=0.378>0.05 of the model construction group and χ^2^ = 8.120, P=0.320>0.05 of the model validation group indicated that the model had high prediction accuracy.

**Originality/value:**

The risk prediction model for CRF holds significant clinical value. It can help medical staff to promptly identify high-risk patients, develop personalized intervention strategies, alleviate fatigue symptoms, and improve overall patient quality of life.

## 1 Introduction

As one of the common malignant tumors in clinical practice, lung cancer originates from lung glands or bronchial mucosa (1–3). According to the latest cancer epidemiology data from China for 2022, lung cancer has the highest incidence and mortality rates among both men and women. In 2022, there were 1.0606 million new cases of lung cancer, accounting for 22.0% of all new malignant tumors, and 733,300 deaths, representing 28.5% of all malignant tumors-related deaths (4). It is worth noting that Galvez Nino et al. (5) observed a significant decrease in the median age of diagnosis from 70 years to 36 years, indicating a troubling trend toward younger age groups. This shift emphasizes the urgent need for more attention to be paid to the health management of lung cancer survivors.

Cancer-related fatigue (CRF) is a debilitating condition associated with cancer and its treatment, characterized by persistent, subjective, physical, emotional, or cognitive exhaustion that is disproportionate to recent activity levels and cannot be alleviated by rest or sleep (6). Recent research shows that CRF in lung cancer patients is generally moderate, with lower family income, prolonged chemotherapy cycles, poor psychological resilience, sleep disorders, and high anxiety contributing to more severe CRF (7). Therefore, exploring the factors influencing CRF in lung cancer patients and developing a risk prediction model is crucial. Current studies have identified key factors and trajectories affecting perioperative CRF in lung cancer patients. For instance, patients who are overweight or obese or who have low psychological resilience, are more prone to persistent fatigue. Additionally, patients with a history of smoking and inadequate social support are associated with exacerbated fatigue symptoms (8). A study by Aihua et al. (9) further identified several risk factors for CRF in patients undergoing chemotherapy for non-small cell lung cancer, including age ≥ 60 years, female, living alone, TNM stage III-IV, poor sleep quality, depressive mood, more than two chemotherapy sessions, FEV 1% < 70%, anemia, pain, nausea, and vomiting. Developing a risk prediction model based on these risk factors could enhance screening and prevention strategies for high-risk patients. However, existing research on prediction models is limited, with most studies focusing mainly on clinical indicators and insufficiently addressing the comprehensive physical and mental factors affecting CRF in lung cancer patients (10, 11). This study, therefore, aims to address this gap by adopting a holistic approach, using the Rived Piper Fatigue Scale to evaluate the degree of CRF in lung cancer patients from four dimensions: behavior, cognition, sensation, and emotion. This study takes into account 32 potential risk factors, including demographic and sociological characteristics, disease-related information, physiological indicators, sleep quality, and mental health, in developing a practical risk prediction model. The predictive factors of this model are relatively easy to obtain in clinical practice, reducing the workload of medical staff while enabling timely identification of high-risk patients, and providing new insights into effective intervention strategies for reducing CRF in lung cancer patients.

## 2 Materials and methods

### 2.1 Research objects

Convenience sampling was used to select 400 lung cancer patients who met the inclusion criteria and visited the oncology, respiratory medicine, thoracic surgery, and other departments of Dazhou Central Hospital, Sichuan Province, from January 2021 to January 2022. This study has been approved by the Ethics Committee of this hospital, with informed consent from all participants.

### 2.2 Sample size estimation

Based on the sample size estimation method for multiple factor logistic regression analysis and a review of relevant literature, a total of 32 variables were identified, with a sample size of 5-10 times the number of variables. Considering a 20% rate of invalid samples, the study determined that a minimum of 388 cases was necessary. Of these, 70% (272 cases) were allocated to establish the risk prediction model, while 30% (116 cases) were reserved for model validation.

### 2.3 Diagnostic criteria

(1) The diagnostic criteria for lung cancer are based on the “Chinese Medical Association Guidelines for Clinical Diagnosis and Treatment of Lung Cancer (Edition 2018)” (12) (2). The pathological classification of lung cancer adheres to the 2015 World Health Organization (WHO) Classification of Tumors of the Lung, Pleura, Thymus and Heart (13) (3). Staging of lung cancer follows the 8^th^ edition of the UICC staging system phases I-IV (14) (4). The diagnostic criteria for CRF are guided by “Chinese Expert Consensus on the Diagnosis and Treatment of Cancer-Associated Fatigue” (15), and are validated using the Revised Piper Fatigue Scale (PFS-R).

### 2.4 Inclusion criteria (those who meet the following four conditions are included)

(1) Inpatients diagnosed with primary lung cancer or those with a diagnosis of primary lung cancer through clinical histopathology; Age ≥ 18 years (2). Functional status rating criteria (Karnofsky, KPS) ≥ 60 points (3). Diagnosed with CRF (4). Expected survival period greater than 3 months (5). The patient is conscious and voluntarily agrees to participate in the questionnaire survey.

### 2.5 Exclusion criteria (excluding those who meet any of the following criteria)

(1) Secondary lung cancer or concurrent presence of other cancers (2). Patients with severe complications such as heart, liver, kidney, hematopoietic system (3). Patients with cognitive impairment (4). Patients under confidential medical treatment.

### 2.6 Research tool

#### 2.6.1 General information survey form

This form collected data on both general and disease-specific characteristics of lung cancer patients namely:

1. Demographic information: Gender (male/female), Age (≥ 65 years/<65 years), Education Level (primary school/junior high school/vocational school or above), Employment Status (employed/retired), Marital Status (unmarried/married/widowed), Living Situation (living alone/with family), Satisfaction with Housing (poor/average/good), Payment Method (self-funded/medical insurance), Monthly Family Income (≥ 5000 yuan/<5000 yuan), Daily Exercise Frequency (≥ 1 time/<1 time).
2. Clinical Information: Pathological Type (squamous cell carcinoma/adenocarcinoma/small cell lung cancer/other), Clinical Stage (phase I/II/III/IV), Disease Course (in days), Necessity of Surgery (yes/no), Treatment Methods (surgery/radiotherapy/chemotherapy/palliative care).
3. Physiological Indicators: Pain Score (using a digital rating scale, ranging from 0-10 points, where 0 indicates no pain and 10 indicates the most severe pain), Complications (yes/no), Chronic Diseases (yes/no), BMI (kg/m^2^), Erythrocyte (×10^12^/L), Hemoglobin (g/L), Platelets (×10^9^/L), Serum Albumin (g/L), Lymphocyte Count (×10^9^/L), Lymphocyte Ratio (%), Blood Pressure [systolic blood pressure (mmHg)/diastolic blood pressure (mmHg)], Blood Glucose (mmol/L), Potassium Ion Concentration (mmol/L), Magnesium Ion Concentration (mmol/L).

#### 2.6.2 PFS-R

PFS-R consists of 22 items and 4 dimensions (behavior, emotion, sensation, cognition). It is scored using an 11-point Likert scale, where 0 indicates no change and 10 indicates very severe fatigue. The total score is the average of the scores across these 4 dimensions. Fatigue severity is categorized as follows: mild fatigue (0 to 3.3 points), Moderate fatigue (3.4-6.7 points), and severe fatigue (6.8-10 points). The scale has demonstrated high reliability, with a test-retest reliability of 0.98 and a Cronbach’s alpha coefficient of 0.91.

#### 2.6.3 Pittsburgh sleep quality index

PSQI mainly reflects the patients’ sleep status over the past month, comprising 19 items across 7 dimensions. Each dimension is scored on a 4-point scale ranging from 0 to 3. A total score greater than 7 indicates sleep disorders, with higher scores indicating more severe sleep disorders. The scale has a Cronbach’s alpha coefficient of 0.87, indicating good reliability.

#### 2.6.4 Hospital anxiety and depression scale

HADS consists of 14 questions, with odd-numbered questions assessing anxiety and even-numbered questions assessing depression. Each question offers 4 options, scored from 0 to 3. A score >7 indicates suspicious anxiety or depression, with higher scores indicating more severe symptoms. The overall Cronbach’s alpha coefficient for the scale is 0.785, while the anxiety and depression subscales have Cronbach’s alpha coefficients of 0.72 and 0.68, respectively.

### 2.7 Data collection

Before the investigation, we formed a team of investigators, consisting of oncology and nursing experts and researchers, who provided standardized training to the team members. The training comprised two components: theoretical and practical skills training. Theoretical training covered relevant topics on lung cancer and CRF, including concepts, epidemiology, clinical manifestations, identification, and evaluation. Practical skills training focused on determining survey questionnaire items, investigation techniques, and necessary precautions. Only after passing the assessment could the investigation work be carried out to ensure standardization and consistency of the investigation. During the survey, with the patient’s informed consent and voluntary participation, the purpose and significance of this study were explained, and patients were informed of their right to voluntarily complete the questionnaire and withdraw at any time. After obtaining consent, the investigators guided the patients to fill out PFS-R, PSQI, and HADS within 3 days of admission, appropriately explaining the meaning, content, and methods of the questionnaire. Patients were instructed to answer truthfully based on their personal situation without fabrication. For patients with difficulty understanding the survey or with many questions, the investigators explained each item and assisted in completing it. This study used a paper version of the questionnaire or Wenjuanxing for data collection. When collecting questionnaires, investigators checked them on the spot for any missing or incomplete items, and promptly inquired about corrections to reduce invalid questionnaires. Starting in January 2022, data from patients admitted for re-examination were collected only once during hospitalization.

### 2.8 Statistics

This study completed all statistical analyses using SPSS27.0 software. Quantitative data conforming to a normal distribution were represented by ( $\bar{x}$ ± s). Independent sample t-test was used for comparison between two groups, and Pearson correlation analysis was used for correlation analysis. Non-normally distributed quantitative data were represented by M (P25, P75). Mann-Whitney *U* rank sum test was used for inter-group comparisons, and Spearman correlation analysis was used for correlation analysis. Categorical data were expressed in logarithmic form, with group comparison performed using the *χ* ^2^ test. Univariate and multivariate logistic regression analyses were conducted to identify the factors influencing CRF in lung cancer patients and to establish a risk prediction model. The discriminative ability of the model was evaluated using the area under the ROC curve (AUC), sensitivity, specificity, Youden index, and other metrics. The Hosmer Lemeshow fit test was used to validate the calibration of the model, with a significant level of P<0.05 considered statistically significant.

## 3 Results

### 3.1 General information of lung cancer patients

This study included 400 patients, which met the sample size requirements. Based on the proportion of risk prediction model construction and validation, 280 patients were included in the model construction group and 120 patients were included in the model validation group. There was no statistically significant difference (P>0.05) in all clinical data between the model construction group and the model validation group, as shown in **Table 1**. The incidence of CRF in the model construction group was 68.8% (193/280). The study subjects were divided into the CRF group (n=192) and NO-CRF group (n=88) based on the occurrence of CRF. In the CRF group, according to the PFS-R scoring criteria, the mild fatigue rate was 17.7% (34/192), the moderate fatigue rate was 51.0% (98/192) and the severe fatigue rate was 31.3% (60/192).

**Table 1:** The comparison of clinical data between the model construction group and the model validation group.

Project	The Model Construction Group (*n*=280)	The model Validation Group (*n*=120)	*χ* ^2^ (*t*-value)	*P-*Value
Gender [n (%)]			0.120	0.900
Male	145 (51.8)	60 (50.0)		
Female	135 (48.2)	60 (50.0)		
Age [n (%)]			1.350	0.250
≥65 years	87 (31.1)	32 (26.7)		
<65 years	193 (68.9)	88 (73.3)		
Education Level [n (%)]			1.500	0.220
Primary school	52 (18.6)	20 (16.7)		
Junior high school	108 (38.6)	35 (29.2)		
Technical secondary school or above	120 (42.8)	65 (54.2)		
Employment Status [n (%)]			0.780	0.430
Employed	125 (44.6)	55 (45.8)		
Retired	155 (55.4)	65 (54.2)		
Marital Status [n (%)]			2.670	0.100
Unmarried	50 (17.9)	25 (20.8)		
Married	210 (75.0)	80 (66.7)		
Widowed	20 (7.1)	15 (12.5)		
Living Situation [n (%)]			1.020	0.310
Living alone	40 (14.3)	18 (15.0)		
With family	240 (85.7)	102 (85.0)		
Satisfaction with Housing [n (%)]			2.340	0.130
Poor	30 (10.7)	8 (6.7)		
General	140 (50.0)	56 (46.7)		
Good	110 (39.3)	56 (46.7)		
Payment Method [n (%)]			0.450	0.500
Self-funded	90 (32.1)	35 (29.2)		
Medical insurance	190 (67.9)	85 (70.8)		
Monthly Family Income [n (%)]			0.680	0.4100
≥5 000 yuan	100 (35.7)	40 (33.3)		
<5 000 yuan	180 (64.3)	80 (66.7)		
Daily Exercise [n (%)]			0.790	0.380
≥1 time	120 (42.9)	45 (37.5)		
<1 time	160 (57.1)	75 (62.5)		
Pathological Type [n (%)]			1.220	0.270
Squamous cell carcinoma	90 (32.1)	35 (29.2)		
Adenocarcinoma	140 (50.0)	55 (45.8)		
Small cell carcinoma of lung	30 (10.7)	20 (16.7)		
Other types	20(7.1)	10 (8.3)		
Clinical Stage [n(%)]			1.530	0.210
Phase I	60 (21.4)	25 (20.8)		
Phase II	70 (25.0)	30 (25.0)		
Phase III	70 (25.0)	35 (29.2)		
Phase IV		30 (25.0)		
Disease Course(x ± *s*, d)	450 ± 50	470 ± 55	1.050^a^	0.300
Necessity of Surgery [n(%)]			0.980	0.320
Yes	100 (35.7)	50 (41.7)		
No	180 (64.3)	70 (58.3)		
Treatment Method [n(%)]			1.320	0.250
Surgery	100 (35.7)	50 (41.7)		
Radiotherapy	80 (28.6)	30 (25.0)		
chemotherapy	60 (21.4)	20 (16.7)		
Palliative care	40 (14.3)	20 (16.7)		
Pain Score (x ± *s*)	5.6 ± 2.3	5.5 ± 2.4	1.030	0.310
Complications [n (%)]			0.750	0.390
Yes	60 (21.4)	25 (20.8)		
No	220 (78.6)	95 (79.2)		
Chronic Diseases[n(%)]			1.240	0.270
Yes	80 (28.6)	40 (33.3)		
No	200 (71.4)	80 (66.7)		
BMI(x ± *s*, kgm^2^)	23.5 ± 3.2	23.7 ± 3.4	0.220^a^	0.820
Erythrocyte (x ± *s*, ×10^12^L)	4.3 ± 0.8	4.2 ± 0.7	0.450^a^	0.500
Hemoglobin (x ± *s*, gL)	120.0 ± 10.5	119.0 ± 11.3	0.600^a^	0.550
Platelet (x ± *s*, ×10^9^L)	250.0 ± 30.6	249.0 ± 29.8	1.230^a^	0.260
Serum Albumin (x ± *s*, gL)	40.0 ± 5.2	41.0 ± 5.5	0.880^a^	0.340
Lymphocyte Count (x ± *s*, ×10^9^L)	2.5 ± 0.6	2.4 ± 0.5	0.670^a^	0.410
Lymphocyte Ratio (x ± *s*, %)	30.0 ± 4.1	29.0 ± 3.8	0.540^a^	0.460
Systolic Pressure (x ± *s*, mmHg)	120.0 ± 10.5	122.0 ± 11.2	1.120^a^	0.28
Diastolic Pressure (x ± *s*, mmHg)	80.0 ± 7.8	81.0 ± 8.1	1.120^a^	0.280
Blood Glucose (x ± *s*, mmol/L)	5.8 ± 0.9	5.7 ± 1.0	0.910^a^	0.370
Potassium (x ± *s*, mmol/L)	4.1 ± 0.3	4.0 ± 0.4	0.230^a^	0.820
Magnesium (x ± *s*, mmol/L)	0.9 ± 0.1	0.9 ± 0.1	0.150^a^	0.880
PSQI score (x ± *s*)	10.2 ± 2.3	10.1 ± 2.4	-0.453	0.652
HADS score (x ± *s*)	12.5 ± 3.1	12.3 ± 3.2	-0.574	0.566

a represnts *t* value; PSQI, Pittsburgh Sleep Quality Index; HADS, Hospital Anxiety and Depression Scale.

### 3.2 Univariate analysis of factors influencing CRF in lung cancer patients in the model construction group

There was a statistically significant difference (P<0.05) between the CRF group and NO-CRF group with respect to gender, age, education level, living situation, Satisfaction with Housing, daily exercise, clinical stage, disease duration, treatment method, presence of chronic diseases, BMI, hemoglobin, serum albumin, blood glucose, potassium ion concentration, magnesium ion concentration, PSQI score, and HADS score, as shown in **Table 2**.

**Table 2:** Univariate analysis of factors influencing CRF in lung cancer patients in the model construction group.

Project	CRF Group (*n*=192)	NO-CRF Group (*n*=88)	*χ* ^2^(*t*) Value	*P* Value
Gender [n (%)]				
Male	90(46.9)	55(62.5)	6.245	0.012
Female	102(53.1)	33(37.5)		
Age[n (%)]				
≥65 years	75(39.1)	12(13.6)	20.456	<0.001
<65 years	117(60.9)	76(86.4)		
Education Level [n (%)]				
Primary school	50(26.0)	2(2.3)	33.682	<0.001
Junior high school	90(46.9)	18(20.5)		
Technical secondary school or above	52(27.1)	68(77.3)		
Employment status [n(%)]				
Employed	90(46.9)	35(39.8)	1.206	0.272
Retired	102(53.1)	53(60.2)		
Marital Status [n(%)]				
Unmarried	40(20.8)	10(11.4)	2.968	0.227
Married	132(68.8)	65(73.9)		
Widowed	20(10.4)	13(14.8)		
Living Situation [n(%)]				
Living alone	30(15.6)	2(2.3)	10.575	0.001
With family	162(84.4)	86(97.7)		
Satisfaction with Housing [n(%)]				
Poor	25(13.0)	2(2.3)	6.921	0.031
General	100(52.1)	40(45.5)		
Good	67(34.9)	46(52.2)		
Payment Method [n(%)]				
Self-funded	70(36.5)	20(22.7)	3.612	0.057
Medical insurance	122(63.5)	68(77.3)		
Monthly Family Income [n(%)]				
≥5 000 yuan	67(34.9)	33(37.5)	0.167	0.683
<5 000 yuan	125(65.1)	55(62.5)		
Daily Exercise [n(%)]				
≥ 1 time	70(36.5)	50(56.8)	10.249	0.002
< 1 time	122(63.5)	38(43.2)		
Pathological Type [n(%)]				
Squamous cell carcinoma	70 (36.5)	20 (22.7)	5.149	0.076
Adenocarcinoma	92 (47.9)	48 (54.5)		
Small cell carcinoma of lung	20 (10.4)	7 (8.0)		
Other types	10 (5.2)	13 (14.8)		
Clinical Stage [n(%)]				
Phase I	15 (7.8)	45 (51.1)	91.635	<0.001
Phase II	20 (10.4)	50 (56.8)		
Phase III	102 (53.1)	18 (20.5)		
Phase IV	55 (28.7)	0 (0)		
Disease Course (x ± *s*, d)	470 ± 55	300 ± 45	19.782^a^	<0.001
Necessity of Surgery [n(%)]				
Yes	70 (36.5)	30 (34.1)	0.141	0.707
No	122 (63.5)	58 (65.9)		
Treatment Method [n(%)]				
Surgery	40 (20.8)	60 (68.2)	55.917	<0.001
Radiotherapy	80 (41.7)	10 (11.4)		
Chemotherapy	50 (26.0)	5 (5.7)		
Palliative care	22 (11.5)	13 (14.8)		
Pain score (x ± *s*)	6.5 ± 2.1	5.0 ± 1.8	6.527^a^	0.011
Complications [n (%)]				
Yes	40 (20.8)	5 (5.7)	9.007	0.003
No	152 (79.2)	83 (94.3)		
Chronic Diseases [n(%)]				
Yes	90 (46.9)	10 (11.4)	31.132	<0.001
No	102 (53.1)	78 (88.6)		
BMI (x ± *s*, kgm^2^)	25.2 ± 3.4	23.0 ± 3.1	4.818^a^	<0.001
Erythrocyte (x ± *s*, ×10^12^L)	4.3 ± 0.8	4.2 ± 0.7	1.217^a^	0.23
Hemoglobin (x ± *s*, gL)	110.0 ± 12.0	120.0 ± 10.0	7.531^a^	<0.001
Platelet (x ± *s*, ×10^9^L)	260.0 ± 35.2	255.0 ± 32.5	1.021^a^	0.311
Serum Albumin (x ± *s*, gL)	38.0 ± 4.5	41.0 ± 5.2	5.412^a^	<0.001
Lymphocyte Count (x ± *s*, ×10^9^L)	2.4 ± 0.5	2.6 ± 0.6	1.987^a^	0.048
Lymphocyte Ratio (x ± *s*, %)	28.0 ± 4.5	30.0 ± 3.8	3.456^a^	0.067
Systolic Pressure (x ± *s*, mmHg)	120.0 ± 10.5	122.0 ± 11.2	1.120^a^	0.264
Diastolic Pressure (x ± *s*, mmHg)	80.0 ± 7.8	81.0 ± 8.1	1.120^a^	0.264
Blood glucose (x ± *s*, mmol/L)	6.2 ± 1.0	5.5 ± 0.8	6.083^a^	<0.001
Potassium Ion C1 timentration (x ± *s*, mmol/L)	4.0 ± 0.4	4.2 ± 0.3	4.003^a^	<0.001
Magnesium Ion C1 timentration (x ± *s*, mmol/L)	0.85 ± 0.1	0.9 ± 0.1	2.561^a^	0.011
PSQI score (x ± *s*)	12.0 ± 2.5	8.0 ± 1.8	16.134^a^	<0.001
HADS score (x ± *s*)	14.5 ± 3.5	10.0 ± 2.7	11.782^a^	<0.001

a denotes the t-value; CRF, Cancer-related fatigue.

### 3.3 Multivariate logistic regression analysis of factors influencing CRF in lung cancer patients in the model construction group

Multiple logistic regression analysis was conducted using the presence of CRF in lung cancer patients as the dependent variable (0=no, 1=yes) and the statistically significant variables from **Table 2** as independent variables (assigned values are shown in **Table 3**). The results showed that gender, age, education level, living situation, daily exercise, clinical stage, disease duration, treatment method, presence of chronic diseases, BMI, hemoglobin, serum albumin, blood glucose, potassium ion concentration, magnesium ion concentration, PSQI score, and HADS score were significant factors influencing CRF in lung cancer patients (P<0.05), as shown in **Table 4**.

**Table 3:** Multivariate logistic regression analysis of independent variable assignment.

Independent Varible	Assignment
Gender	Femlae=0, Male=1
Age	<65 years =0,≥65 years =1
Education Level	Primary school=0, Junior high school=1, Technical secondary school or above=2
Living Situation	With family=0, living alone=1
Satisfaction with Housing	Poor=0, general=1, good=2
Daliy Excersice	< 1 time=0, ≥1 time=1
Clinical Stage	Phase I=0, phase II=1, phase III=2, phase IV=3
Disease Course	Actual measurement
Treatment Method	Palliative care=0, chemotherapy=1, radiotharapy=2, surgery=3
Complications	No=0, yes=1
Chronic diseses	No=0, yes=1
BMI	Actual measurement
Hemoglobin	Actual measurement
Serum Albumin	Actual measurement
Blood Glucose	Actual measurement
Potassium Ion C1 timentration	Actual measurement
Magnesium Ion C1 timentration	Actual measurement
PSQI score	Actual measurement
HADS score	Actual measurement

**Table 4:** Multivariate logistic regression analysis of the factors influencing CRF in lung cancer patients in the model construction group.

Varible	*B*	*SE*	Wald *χ* ^2^ Value	*P* Value	*OR* Value	95%*CI*
Gender (with reference to male)						
Female	0.762	0.321	5.629	0.018	2.142	1.142-4.019
Age (with reference to <65 years)						
≥65 years	1.292	0.411	9.884	0.002	3.640	1.597-8.307
Education Level(with reference to primary school)						
Junior high school	-0.543	0.223	5.928	0.015	0.581	0.375-0.900
Technical secondary school or above	-1.012	0.322	9.886	0.002	0.364	0.190-0.697
Living Situation(with reference to with family)						
Living Alone	1.082	0.484	4.999	0.025	2.950	1.143-7.617
Satisfaction with Housing (with reference to poor)						
General	0.281	0.466	0.363	0.547	1.324	0.529-3.318
Good	-0.241	0.487	0.245	0.621	0.786	0.300-2.060
Daily Exercise(with reference to < 1 time)						
≥ 1 time	0.731	0.334	4.796	0.029	2.078	1.078-4.004
Clinical Stage(with reference to phase I)						
Phase II	0.942	0.556	2.869	0.090	2.566	0.859-7.664
Phase III	1.632	0.494	10.909	0.001	5.114	1.936-13.504
Phase IV	2.484	0.525	22.367	<0.001	11.986	4.334-33.153
Disease Course	0.01	0.003	10.958	0.001	1.01	1.004-1.016
Treatment Method (with reference to palliative care)						
Chemotherapy	1.011	0.576	3.085	0.079	2.748	0.895-8.442
Radiotherapy	1.959	0.636	9.489	0.002	7.092	2.065-24.349
Surgery	2.251	0.692	10.579	0.001	9.494	2.433-37.070
Complications (with reference to no)						
Yes	0.682	0.620	1.211	0.271	1.978	0.580-6.746
Chronic Diseases (with reference to no)						
Yes	1.624	0.536	9.154	0.002	5.072	1.764-14.575
BMI	0.186	0.074	6.304	0.012	1.204	1.041-1.392
Hemoglobin	-0.042	0.014	8.749	0.003	0.959	0.932-0.986
Serum Albumin	-0.071	0.031	5.306	0.021	0.931	0.876-0.989
Blood Glucose	0.473	0.145	10.679	0.001	1.605	1.201-2.146
Potassium Ion C1 timentration	-1.845	0.667	7.659	0.006	0.158	0.043-0.574
Magnesium Ion C1 timentration	-2.128	0.905	5.530	0.019	0.119	0.018-0.759
PSQI Score	0.365	0.086	18.061	<0.001	1.440	1.218-1.703
HADS Score	0.442	0.086	26.536	<0.001	1.556	1.317-1.839

According to the results of multiple logistic regression analysis, the risk prediction model for CRF in lung cancer patients can be expressed by the following equation:

logit (P)=0.762 × gender+1.292 × age -0.543 × junior high school education level -1.012 × technical secondary school education level or above+1.082 × living alone+0.731 × daily exercise status+0.942 × phase II +1.632 × phase III +2.484 × phase IV +0.010 × disease course+1.011 × chemotherapy+1.959 × radiotherapy+2.251 × surgery+1.624 × chronic disease+0.186 × BMI -0.042 × hemoglobin -0.071 × serum albumin+0.473 × blood glucose -1.845 × potassium ion concentration -2.128 × magnesium ion concentration+0.365 x PSQI score+0.442 x HADS score.

### 3.4 Analysis of the predictive effect of CRF prediction model for lung cancer patients

**Figure 1:**
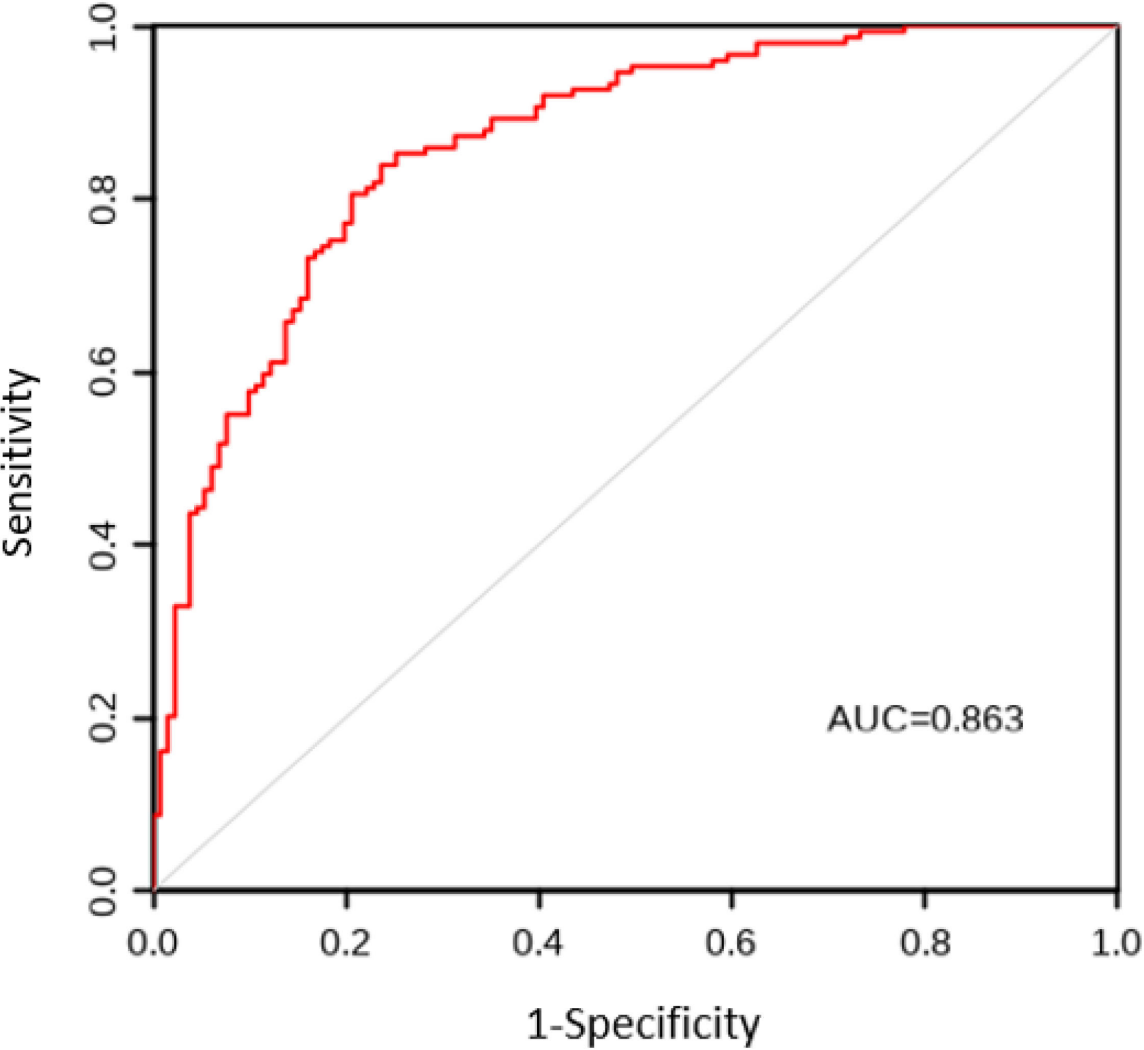
ROC curve of CRF prediction model for lung cancer patients in the model construction group. ROC, Subjects’ Functional Curve; AUC, Area Under ROC Curve; CRF, Cancer-related Fatigue.

**Figure 2:**
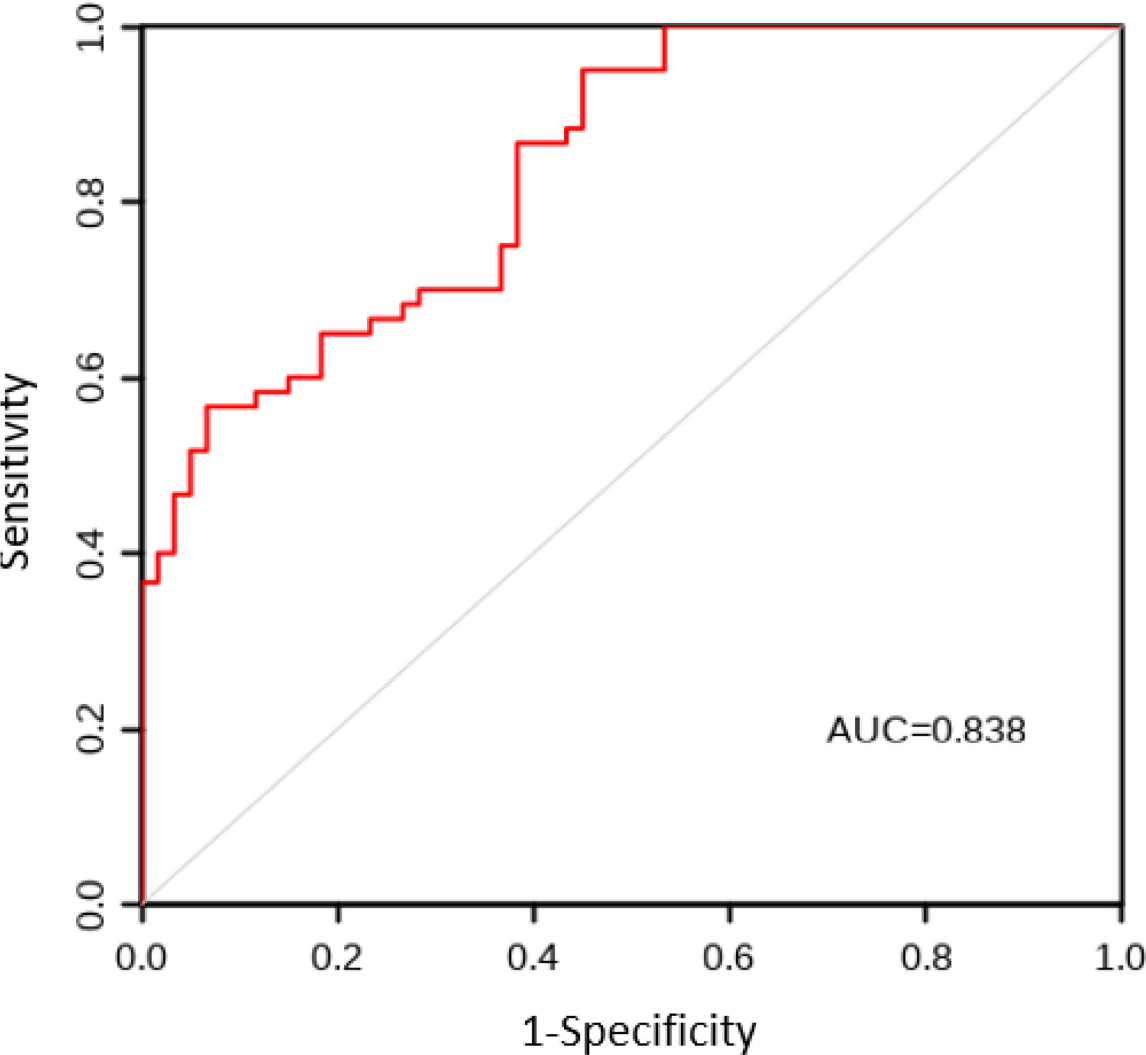
ROC curve of CRF prediction model for lung cancer patients in the model validation group.

The AUC values for the model construction group and the model validation group were 0.863 and 0.838, respectively. The sensitivity, specificity, and Youden index results are shown in **Table 5**, with the ROC curves shown in **Figures 1** and **2**. The Hosmer Lemeshow fitting test results indicated that the model construction group had a χ^2^ value of 7.54, P=0.378>0.05, while the model validation group had a χ^2^ value of 8.12, P=0.320>0.05. These results suggest no statistically significant difference between the predicted values and the actual measurement of the model, indicating that the prediction model has good calibration ability.

**Table 5:** Diagnostic value of CRF prediction model in model construction group and model validation group.

Group	*AUC*	95%*CI*	Sensetivity(%)	Specificity (%)	Youden Index	*P* Value
Model Construction Group	0.863	0.812-0.902	82.50	78.30	0.608	<0.001
Model Validation Group	0.838	0.784-0.898	80.20	76.50	0.567	<0.001

## 4 Discussions

CRF is one of the common adverse reactions in the treatment of cancer patients, which seriously affects their quality of life and treatment effectiveness (16). The mechanism of CRF is complex and involves multiple biological and psychological factors (17). In recent years, an increasing number of studies have explored the biological mechanisms of CRF, including anemia, abnormal cytokine regulation, abnormal hypothalamic pituitary adrenal axis regulation, abnormal regulation of 5-hydroxytryptophan neurotransmitter, and changes in ATP and muscle metabolism (18).The most prominent hypothesis among scholars is the dysregulation of pro-inflammatory cytokines, known as the inflammation hypothesis (19, 20). However, there is still no unified understanding of the exact pathogenesis of CRF.

The occurrence and development of CRF are influenced by multiple factors (21). Weis et al. (22) found that 59% to 100% of cancer patients experience CRF. Common cancer treatments such as surgery, radiotherapy, chemotherapy, and biological therapy have varying degrees of impact on CRF (23). Although CRF may occur before cancer treatment, it typically worsens during radiotherapy, chemotherapy, surgery, or biological therapy. Berger et al. (24) reported that cancer patients undergoing radiotherapy, chemotherapy, and combined treatments had CRF incidence rates of 30% to 91%, 25% to 93%, and 59% to 93%, respectively. However, research into the factors influencing CRF in liver cancer patients remains scarce. In this study, variables such as gender, age, educational level, living situation, daily exercise habits, clinical stage, disease course, treatment method, presence of chronic conditions, BMI, hemoglobin levels, serum albumin, glucose, potassium ion concentration, magnesium ion concentration, PSQI score, and HADS score were incorporated into a multivariate logistic regression analysis to ascertain their influence on CRF in lung cancer patients.

Firstly, gender significantly affects CRF, with female patients being more likely to develop CRF than male patients (25). This may be related to women being more sensitive to pain and fatigue, and having poorer psychological resilience. Yan Wenjing et al. (26) found in a cluster analysis of 220 hospitalized lung cancer chemotherapy patients that female patients exhibited fatigue, negative emotion, pain, and sleep disorder syndrome more frequently. Age is also a significant factor, with older patients (≥ 65 years old) more likely to develop CRF. Elderly patients with decreased physical function are more prone to malnutrition and anemia, coupled with heavy psychological burden and more pronounced fatigue (27). Jacobsen et al. (28) found that the incidence and severity of fatigue in cancer patients with hemoglobin levels<12 g/dL after chemotherapy were significantly positively correlated with hemoglobin levels.

Moreover, the influence of education level on CRF cannot be ignored. Patients with a junior high school education or below are more likely to experience fatigue than those with a technical secondary school education or above. This may be due to lower levels of education and poorer understanding and coping abilities with the disease, as well as insufficient social support (29). Patients living alone are more likely to feel lonely and fatigued due to a lack of family support and social interaction (30). The relationship between daily exercise and CRF has also been validated, with patients with lower exercise frequency having a higher incidence of CRF (31). Appropriate exercise can enhance the body’s immune system, improve mood, and reduce fatigue.

Clinical stage is another important factor, and patients with later staging (phases III-IV) have a higher incidence of CRF. This may be due to the severe condition, high treatment intensity, and heavy physical and psychological burden of late-stage patients (32, 33).

In terms of treatment methods, patients who receive chemotherapy and radiotherapy have a higher incidence of CRF. Although these treatment methods are effective, they have significant side effects that lead to physical weakness and increased fatigue in patients (34). In addition, the presence of chronic diseases increases the risk of CRF. Patients with chronic diseases, due to the presence of underlying conditions experience further limitations in their physical functions, making them more likely to feel tired (35). Physiological indicators such as BMI, hemoglobin, serum albumin, blood glucose, potassium ion concentration, and magnesium ion concentration are also closely related to CRF. Patients with low hemoglobin and serum albumin levels are more prone to fatigue due to the decreased ability of the body to transport oxygen and nutrients. (36) Hypoglycemia and electrolyte imbalances, such as low potassium and magnesium concentrations can also exacerbate fatigue (37). The impact of sleep quality and mental health status on CRF is particularly significant. Patients with poor sleep quality experience more pronounced fatigue due to their inability to recover their energy through sleep. (38)Anxiety and depression can also exacerbate the symptoms of CRF (39). Multiple studies have demonstrated a close relationship between depression and fatigue among the negative emotions affecting CRF. Research by LIN and other researchers has shown that pain, sleep disorders, negative emotions, and CRF interact with each other, leading to an increase in the incidence of CRF in cancer patients and significantly affecting their quality of life (40, 41).

In summary, the occurrence of CRF in lung cancer patients results from multiple interacting factors. Significant factors include gender, age, education level, living situation, daily exercise frequency, clinical stage, disease course, treatment methods, chronic diseases, BMI, hemoglobin, serum albumin, blood glucose, potassium ion concentration, magnesium ion concentration, sleep quality, and mental health status. The risk prediction model constructed based on these factors has important application value in clinical practice. It can help medical staff identify high-risk patients promptly, formulate personalized intervention strategies, alleviate fatigue symptoms, and improve patients’ quality of life.

While this study contributes to addressing an important research gap and offers significant clinical application value, it is not without limitations. Sample size constraints and inherent biases in observational study design may affect the generalizability of the results. Additionally, the limited time points for data collection prevented us from fully capturing all the dynamic factors that are likely to affect the development of CRF, This study only reported short-term effects and lacked long-term follow-up of patients, which is crucial for assessing the long-term effects of cancer-related fatigue. Future research should, therefore, consider larger-scale surveys and incorporating objective indicators, such as biomarkers to further validate the effectiveness and accuracy of the predictive model. Cancer-related fatigue is an important research area, more high-quality, multicenter, standardized studies are needed to provide more effective treatment strategies, exploring the specific mechanisms of CRF across different populations and its impact on personalized intervention measures will provide a more robust evidence base and support for CRF management in lung cancer patients, to improve the quality of life of cancer patients and prolong the overall survival.

## Data Availability

The datasets presented in this study can be found in online repositories. The names of the repository/repositories and accession number(s) can be found in the article/supplementary material.
